# Impact of Web-Based Cognitive Behavioral Therapy for Insomnia on Stress, Health, Mood, Cognitive, Inflammatory, and Neurodegenerative Outcomes in Rural Dementia Caregivers: Protocol for the NiteCAPP CARES and NiteCAPP SHARES Randomized Controlled Trial

**DOI:** 10.2196/37874

**Published:** 2022-06-14

**Authors:** Christina S McCrae, Ashley F Curtis, Amelia Cottle, David B Beversdorf, Joel Shenker, Brian P Mooney, Mihail Popescu, Marilyn Rantz, Maureen Groer, Phyllis Stein, Mojgan Golzy, Melanie A Stearns, Angelynn Simenson, Neetu Nair, Meredeth A Rowe

**Affiliations:** 1 University of Missouri Columbia, MO United States; 2 University of South Florida Tampa, FL United States; 3 Washington University St Louis, MO United States

**Keywords:** caregiver, CBT-I, cognition, dementia, inflammation, insomnia, neurodegeneration

## Abstract

**Background:**

Chronic insomnia affects up to 63% of family dementia caregivers. Research suggests that chronic insomnia prompts changes in central stress processing that have downstream negative effects on health and mood, as well as on cognitive, inflammatory, and neurodegenerative functioning. We hypothesize that cognitive behavioral therapy for insomnia (CBT-I) will reverse those downstream effects by improving insomnia and restoring healthy central stress processing. Rural caregivers are particularly vulnerable, but they have limited access to CBT-I; therefore, we developed an accessible digital version using community input (NiteCAPP CARES).

**Objective:**

This trial will evaluate the acceptability, feasibility, and short-term and long-term effects of NiteCAPP CARES on the sleep and stress mechanisms underlying poor caregiver health and functioning.

**Methods:**

Dyads (n=100) consisting of caregivers with chronic insomnia and their coresiding persons with dementia will be recruited from Columbia and surrounding areas in Missouri, United States. Participant dyads will be randomized to 4 weeks (plus 4 bimonthly booster sessions) of NiteCAPP CARES or a web-based sleep hygiene control (NiteCAPP SHARES). Participants will be assessed at baseline, after treatment, and 6- and 12-month follow-ups. The following assessments will be completed by caregivers: 1 week of actigraphy and daily diaries measuring sleep, Insomnia Severity Index, arousal (heart rate variability), inflammation (blood-derived biomarkers: interleukin-6 and C-reactive protein), neurodegeneration (blood-derived biomarkers: plasma amyloid beta [Aβ40 and Aβ42], total tau, and phosphorylated tau [p-tau181 and p-tau217]), cognition (Joggle battery, NIH Toolbox for Assessment of Neurological and Behavioral Function, and Cognitive Failures Questionnaire), stress and burden, health, and mood (depression and anxiety). Persons with dementia will complete 1 week of actigraphy at each time point.

**Results:**

Recruitment procedures started in February 2022. All data are expected to be collected by 2026. Full trial results are planned to be published by 2027. Secondary analyses of baseline data will be subsequently published.

**Conclusions:**

This randomized controlled trial tests NiteCAPP CARES, a web-based CBT-I for rural caregivers. The knowledge obtained will address not only what outcomes improve but also how and why they improve and for how long, which will help us to modify NiteCAPP CARES to optimize treatment potency and support future pragmatic testing and dissemination.

**Trial Registration:**

ClinicalTrials.gov NCT04896775; https://clinicaltrials.gov/ct2/show/NCT04896775

**International Registered Report Identifier (IRRID):**

PRR1-10.2196/37874

## Introduction

### Background

Approximately 16 million Americans serve as unpaid informal caregivers (CGs), providing 18.6 billion hours of care, which translates into US $244 billion in health care savings [1]. The number of persons with dementia in the United States is projected to rise from 5.7 million to >14 million in the next 30 years, and most persons with dementia (70%) are cared for at home by a family member [1]. This rise in the number of persons with dementia is likely to increase the number of CGs, especially in rural areas, where populations are aging faster than those in urban areas [2]. Chronic insomnia (≥3 months difficulty initiating and maintaining sleep, early morning awakening, or nonrestorative sleep) affects up to 63% of CGs [3,4] and tends to endure (18 years on average in our CG studies) [4,5]. Factors associated with CG insomnia include changes in central stress processing (increased sympathetic nervous system arousal) [3,6] as well as worse health and daytime functioning (increased depression, stress, and cognitive dysfunction). Research [3-5] shows that cognitive behavioral therapy for insomnia (CBT-I) is an efficacious treatment for the chronic and particularly long-lasting insomnia experienced by CGs and suggests that it may restore healthy central stress processing [7]. However, rural CGs have less access to care, including CBT-I, because of significant shortages of both primary and specialty care providers in rural areas [8]. Web-based CBT-I will allow for greater dissemination, particularly for rural CGs and persons with dementia who may have challenges receiving treatment because of trained provider scarcity [9] and the burden associated with traditional in-office delivery.

It has been shown by our team as well as another group of researchers that in-person brief CBT-I (bCBT-I) improves CG sleep and mood (small to large effects) [10-12]. Early research by McCurry et al [12] found that in-person bCBT-I improved CG sleep (efficiency and quality) and depression immediately and 3 months after treatment compared with a waitlist. Our team also found that in-person bCBT-I improved sleep, depression, and anxiety in older adult CGs of persons with dementia [10]. McCrae et al [11] also found that bCBT-I (2 in-person sessions and 2 telehealth sessions) improved sleep in rural older adults for at least 3 months compared with the sleep hygiene control. Our new pilot data also suggest that CBT-I translates well to telehealth [7,13]. However, telehealth delivery still requires considerable time commitment from trained therapists (already in short supply). Thus, web delivery represents an important, logical next step that would maximize therapist time (ie, web-based moderation and feedback vs 1 hour per week one-on-one sessions), reduce CG travel burden, and give both therapist and CG scheduling flexibility. Web delivery would also enhance disseminability because the majority of Americans (including rural and older Americans) use the internet and have access to it [14]. Moreover, several meta-analyses of web-based CBT-I have indicated moderate to large effect sizes for sleep improvement that are comparable to in-person CBT-I effects [15,16]. Although web-based CBT-I has not been tested in CGs, web interventions have successfully improved noninsomnia health outcomes in stroke and CGs [7,17]. These findings provide support for the development and evaluation of web-based CBT-I for CGs (NiteCAPP CARES) in the proposed study.

Several considerations were taken into account in the development of NiteCAPP CARES. For instance, given prior work showing that providing CGs with behavioral sleep instructions for their persons with dementia led to improved sleep in persons with dementia (measured using actigraphy) [7], NiteCAPP CARES uses a dyadic approach that targets sleep for both the CG and care recipient. In addition, following recommendations from other CBT-I studies [16], NiteCAPP CARES uses a guided therapy approach with trained therapy moderators monitoring the progress of CGs and persons with dementia, providing feedback, and answering questions. Taking a dyadic, guided therapy approach may optimize the impact of CBT-I on CG mechanisms and outcomes.

Research suggests that CG chronic insomnia results in changes in central stress processing that have downstream negative effects on health and daytime functioning. CGs experience increased subjective stress and arousal because they witness the distress experienced by persons with dementia and because of the physical and time demands of caregiving [4,5]. Research shows that persons with chronic insomnia have alterations in heart rate (HR) and HR variability (HRV) while awake before sleep and during stage 2 non–rapid eye movement sleep [18], as well as lower wake-to-sleep HR reduction and SD of normal-to-normal heartbeat intervals compared with controls [19], consistent with disrupted autonomic nervous system (ANS) functioning (increased sympathetic activity). In CGs, increased self-reported stress is associated with altered HRV during the first half of sleep (lower high-frequency power values), indicative of impaired ANS functioning [19]. Our preliminary results have shown that HRV can be altered because bCBT-I increased resting HRV in adults with comorbid insomnia [10]. Collectively, the findings in comorbid insomnia and our preliminary data suggest that physiological arousal serves as a mechanism underlying other CG symptoms.

CGs also face mental and physical health–related challenges regarding mood and cognition, as well as neurodegenerative and inflammatory biomarkers. Sleep may play a role in CGs’ high levels of anxiety and depression [20] because our research shows that nights of poor sleep (ie, higher total wake time and poorer sleep quality) were associated with greater next-day negative affect in CGs [10]. Research, including ours, shows that CBT-I can improve depression in CGs [12,21,22]. In addition, CGs perform worse on tests of processing speed [23,24], attention [23,25,26], executive function [26], and memory [26,27] than same age non-CGs. Our recent trial shows that telehealth CBT-I can improve CG executive functioning and processing speed [10]. Moreover, preclinical studies link poor sleep to increased Alzheimer disease–related biomarkers (plasma amyloid beta [Aβ] and tau), suggesting that sleep is essential for protein clearance and reduction of neuronal aggregations contributing to plaques and tangles (hallmark pathology of Alzheimer disease) [27]. Risk of developing Alzheimer disease is elevated in CGs versus non-CGs (up to 6 times greater) [28], and CGs have increased peripheral inflammatory biomarker levels (eg, C-reactive protein) [29]. Sleep quality improvements are associated with reductions in inflammatory markers associated with Alzheimer disease, that is, C-reactive protein (small effects) and interleukin-6 concentrations (large effects) [29], as well as expression of inflammatory-related genes [30]. These studies suggest that targeting poor sleep may lower Alzheimer disease–related biomarker levels, thereby mitigating risk and delaying disease progression. The proposed trial will examine whether treating CGs’ sleep disturbances through NiteCAPP CARES may help mitigate their neurodegenerative risks.

**Figure 1:**
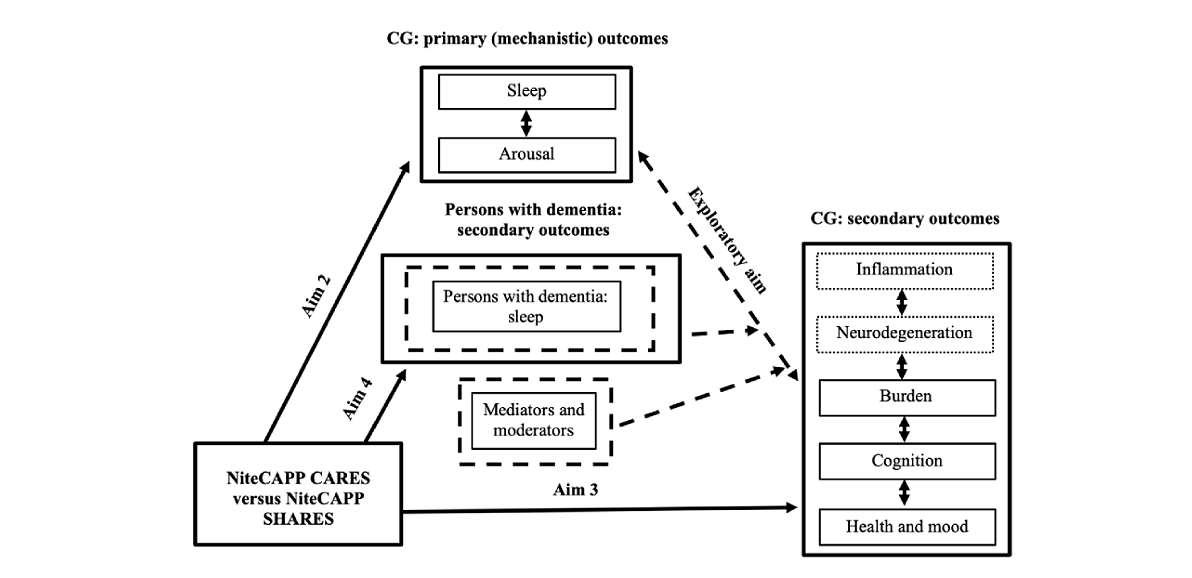
Conceptual model. Aim 1 (not shown) examines feasibility and acceptability of NiteCAPP CARES and NiteCAPP SHARES. Sleep change in person with dementia will be examined as an outcome (aim 4) and a potential moderator (exploratory aim: dashed lines) of the relationship between changes in caregiver (CG) primary and secondary outcomes. Refer to the Outcomes section for a list of potential mediators (eg, adherence) and moderators (eg, sleep change in person with dementia, interpersonal processes, and shared lifestyle factors). Biomarkers of neurodegeneration and inflammation will be examined as exploratory CG secondary outcomes (dotted box outline). NiteCAPP CARES: cognitive behavioral therapy for insomnia; NiteCAPP SHARES: active web-based sleep hygiene and related education control.

The Cognitive Activation Theory of Stress [31] underlies the basic premise guiding the proposed trial, namely that CGs experience chronic insomnia, arousal, and inflammation that are associated with central nervous system changes that negatively affect other CG health outcomes (Figure 1). Specifically, arousal, poor sleep, and inflammation lead to critical changes in the hypothalamic-pituitary-adrenal axis and ANS, primarily sympathetic activation functioning, that contribute to lower health-related quality of life, increased depression and burden, and decreased cognitive functioning. We propose that NiteCAPP CARES holds promise for improving multiple CG health outcomes by improving common underlying mechanisms (sleep and arousal), thereby restoring healthy hypothalamic-pituitary-adrenal axis and ANS functioning. NiteCAPP CARES will engage these mechanisms using established sleep techniques (sleep education, sleep hygiene, and stimulus control), modified techniques (for older adults and CGs; sleep compression and brief relaxation), techniques for other CG issues (problem solving and stress management), and by targeting sleep change in persons with dementia (direct participation of persons with dementia or CG administration of behavioral techniques).

The base NiteCAPP platform was developed in 2019 by the study team and was based on a 4-session CBT-I treatment protocol developed by the first author (CSM) and used for research [10,32] and clinical service delivery for >20 years. Initial adaptations for CGs to create NiteCAPP CARES were made following the Medical Research Council recommendations for evaluating complex medical interventions, including community participation, usability testing, and validation. The details of the preliminary work are available elsewhere [7]. Although the findings from these usability studies were promising and demonstrated the preliminary feasibility and acceptability of NiteCAPP CARES as well as its efficacy for improving sleep and other variables for CGs, they warrant further investigation in a larger sample and comparison with an active control condition across a wider range of outcomes. The proposed trial offers the following methodological improvements: inclusion of an active control group (web-based sleep hygiene and related education [NiteCAPP SHARES]), increased number and length of follow-ups, and a dyadic approach (because persons with dementia experience poor sleep and nighttime behavior that negatively affects CGs). In sum, the proposed trial addresses significant gaps in the literature and the need for effective insomnia treatment for CGs and persons with dementia.

### Objectives

The overarching goal of this randomized controlled trial (RCT) is to evaluate the acceptability, feasibility, and short-term and long-term effects of NiteCAPP CARES on the sleep and stress mechanisms underlying poor CG health and functioning. Our first specific aim is to examine the feasibility and acceptability of NiteCAPP CARES and an active web control (sleep hygiene and related education [NiteCAPP SHARES]). We hypothesize that NiteCAPP CARES and NiteCAPP SHARES will have high rates of completion (≥75% of sessions), adherence (≥75% of the techniques implemented), satisfaction (≥7.5 out of 10 on the Satisfaction Survey), and utility (≥3.5 out of 5 on the Internet Intervention Utility Questionnaire).

Our second specific aim is to examine the effects of NiteCAPP CARES on the primary (mechanistic) CG outcomes in our theory-driven conceptual model; that is, to examine the effects of 4 weeks of CBT-I compared with 4 weeks of NiteCAPP SHARES on CG sleep (insomnia severity and self-reported sleep onset latency, wake time after sleep onset, and sleep efficiency) and arousal immediately after treatment and at 6- and 12-month follow-ups. We hypothesize that compared with NiteCAPP SHARES, NiteCAPP CARES will improve CG sleep and decrease arousal.

Our third specific aim is to examine the effects of NiteCAPP CARES on the secondary CG outcomes in our theory-driven conceptual model; that is, to examine the effects of 4 weeks of CBT-I compared with weeks of NiteCAPP SHARES on CG health, mood, burden, cognition, inflammation, and neurodegeneration immediately after treatment and at 6- and 12-month follow-ups. We hypothesize that compared with NiteCAPP SHARES, NiteCAPP CARES will improve these CG outcomes.

Our fourth specific aim is to test the effect of NiteCAPP CARES on persons with dementia, the secondary outcome in our theory-driven conceptual model; that is, to examine the effects of 4 weeks of CBT-I compared with 4 weeks of NiteCAPP SHARES on sleep change in persons with dementia (objective measurement through actigraphy and total sleep time) immediately after treatment and at 6- and 12-month follow-ups. We hypothesize that compared with NiteCAPP SHARES, NiteCAPP CARES will improve sleep in persons with dementia.

Our exploratory aim is to examine the relationships between CG primary and secondary outcome changes and their potential mediators (eg, adherence) and moderators (eg, sleep change in persons with dementia, interpersonal processes, and shared lifestyle factors).

## Methods

### Trial Design and Study Setting

Dyads (n=100) consisting of CGs with chronic insomnia and their coresiding persons with dementia will be recruited from Columbia and surrounding areas in Missouri, United States, and randomized to 4 sessions of NiteCAPP CARES or NiteCAPP SHARES. The groups will receive bimonthly boosters. Baseline and posttreatment assessments as well as 6-month and 12-month follow-ups will measure sleep, arousal, biomarkers of inflammation and neurodegeneration, health, mood, burden, and cognition.

Graduate therapists and assessors will obtain written informed consent from CGs and persons with dementia, if possible. If the person with dementia is unable to consent (determined by consultation with the first author [CSM]), a legally authorized representative must sign a consent form on their behalf. If the person with dementia becomes unable to provide consent during the course of the study, the legally authorized representative will be asked to reconsent on behalf of the care recipient. Dyads in both groups will receive US $125 at baseline, US $150 after treatment, US $175 at 6-month follow-up, and US $200 at 12-month follow-up, as well as treatment at no cost. NiteCAPP SHARES participants will be offered NiteCAPP CARES at no cost after the study.

### Eligibility Criteria

The CG inclusion criteria are as follows: (1) aged ≥18 years, (2) CG living with persons with dementia, (3) willing to be randomized, (4) able to read and understand English, (5) diagnosed with insomnia [33-35], and (6) no newly prescribed or over-the-counter sleep medications for ≥1 month or stabilized for ≥6 months. Insomnia diagnosis requires (1) sleep complaints for ≥6 months, (2) adequate opportunity and circumstances for sleep, (3) at least one of the following: difficulty falling asleep or staying asleep or waking too early, (4) daytime dysfunction (mood, cognitive, social, or occupational) because of insomnia, and (5) baseline diaries indicate >30 minutes of sleep onset latency or wake after sleep onset on ≥3 nights.

The CG exclusion criteria are as follows: (1) unable to consent, (2) cognitive impairment (based on score of ≤25 on the Montreal Cognitive Assessment), (3) sleep disorder other than insomnia (ie, apnea (Apnea-Hypopnea Index score>15), (4) bipolar or seizure disorder, (5) other major psychopathology except depression or anxiety (eg, suicidal or psychotic), (6) severe untreated psychiatric comorbidity, (7) psychotropic or other medications (eg, clonidine) that alter sleep, and (8) nonpharmacological treatment for sleep or mood outside of current trial.

The inclusion criteria for persons with dementia are as follows: (1) probable or possible Alzheimer disease (self-report or primary care physician’s written confirmation), (2) at least one problem on the Nighttime Behavior Inventory ≥3 nights per week, (3) able to tolerate actigraphy, (4) not taking any new sleep medications for ≥1 month or stabilized ≥6 months, (5) untreated sleep disorder for which CBT-I is not recommended (ie, sleep apnea), and (6) scoring <32 on the sleep apnea scale on the Sleep Disorders Questionnaire.

### Randomization and Blinding

Computer randomization will be performed by the team’s biostatistician, using a permuted block randomization technique stratified by baseline sleep medication use (yes or no) [36]. Other personnel (except for therapists, supervisor, and project coordinator) will be blinded to randomization.

### Procedures

#### Screening

A sleep psychologist (principal investigator CSM) will diagnose insomnia and make referrals for other suspected sleep disorders (eg, apnea). Screening will be carried out in four stages:

- Stage 1: brief screener (15 minutes). The project coordinator will (1) conduct a brief structured interview to address the inclusion and exclusion criteria and establish a probable insomnia diagnosis and (2) administer the Montreal Cognitive Assessment (CG must score >25, a cutoff chosen to maintain consistency with our prior clinical trial).
- Stage 2: clinical interview (50 minutes). The assessor will (1) conduct a semistructured sleep and psychiatric interview and (2) facilitate and assist with web-based baseline questionnaire completion.
- Stage 3: apnea testing (1 overnight session). This consists of 1 night of at-home sleep testing using disposable WatchPAT One devices (Itamar Medical Ltd) to rule out sleep disorders other than insomnia (ie, apnea). The assessor will instruct CGs on how to use WatchPAT One devices in their own homes so that the CGs can sleep in their own beds. Referrals will be made for those disqualified. The WatchPAT One (a wrist-worn device with 2 finger sensors) measures peripheral arterial tone, oximetry, actigraphy, HR, body position, and snoring.
- Stage 4: sleep diary confirmation of insomnia (5 minutes per day). Electronic baseline diaries will confirm insomnia diagnosis and must show >30 minutes of sleep onset latency or wake after sleep onset on ≥3 nights over 7 days. Diaries will be collected electronically through Qualtrics software with personal web-enabled devices or (if needed) study-provided devices and internet service.

#### Interventions

**Figure 2:**
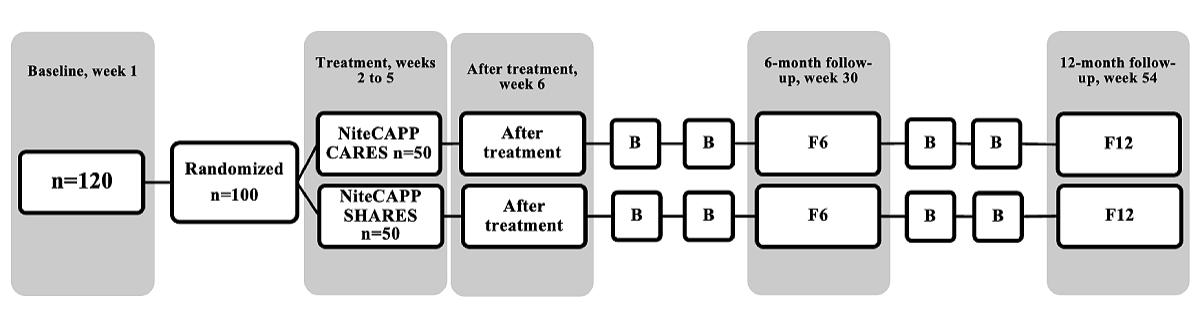
Session and booster timeline. The randomization numbers refer to dyads (ie, caregivers and their coresiding persons with dementia). B: booster session; F6: 6-month follow-up; F12: 12-month follow-up; NiteCAPP CARES: cognitive behavioral therapy for insomnia; NiteCAPP SHARES: active web-based sleep hygiene and related education control.

Both arms (NiteCAPP CARES and NiteCAPP SHARES) include 4 web-based sessions and 4 bimonthly, 20-minute boosters (Figure 2) with a therapist moderator (predoctoral graduate students in an American Psychological Association–accredited psychology program at the University of Missouri). Details of the development of NiteCAPP have been published and are available [7]. Each session is completed individually by CGs (with persons with dementia to the extent able) in a single sitting (<45 minutes). Each session should be completed in 7 days, with the next session released only after the previous one is completed. All procedures will be conducted at participants’ homes. Session content details for NiteCAPP CARES and NiteCAPP SHARES are provided in Textboxes 1 and 2, respectively.

Both interventions will be dyadic, and CGs will work with persons with dementia to implement behavioral strategies similar to those used by McCrae et al [10] and McCurry et al [12]. Active participation of persons with dementia is a goal but may be limited because of dementia symptoms. Behavioral strategies for persons with dementia will be tailored based on baseline assessment (use of actigraphy in persons with dementia, nighttime behaviors, and dementia severity and other questionnaires). To provide a guided therapy approach, trained moderators (psychology doctoral students) will (1) monitor adherence and progress (log-in details and log completion), (2) provide weekly email feedback on sleep patterns and progress, (3) answer questions within 24 hours through a web-embedded message forum, and (4) provide bimonthly boosters (discuss maintenance and troubleshoot problems).

**Session content for NiteCAPP CARES.**

**1. Sleep hygiene, sleep education, and stimulus control**

- Sleep hygiene will be taught, and participants are instructed to adhere to the following rules (the goal of sleep hygiene is to eliminate sleep-interfering behaviors):
  avoid caffeine after noon
  within 2 hours of going to bed, avoid exercise and heavy meals
  within 1 hour of bedtime, avoid screen time
  use the bed for sleeping only

**2. Sleep compression, relaxation, and problem solving**

- The therapist moderator and caregiver and person with dementia will work together to create a sleep prescription and set regular bed and wake times consistent with the prescription. Caregiver and person with dementia will be instructed to practice relaxation techniques. Caregiver will be provided suggestions on how to solve the challenges associated with caregiving for a person with dementia.

**3. Coping and stress management and cognitive therapy**

- Sleep prescription for caregiver and person with dementia will be updated as appropriate. Coping and stress management techniques will be taught. Caregiver and person with dementia will be taught how to identify maladaptive thoughts and replace them with balanced thoughts.

**4. Review and education and plan for maintenance of behavior change**

- Sleep prescription for caregiver and person with dementia will be updated as appropriate. Caregiver and person with dementia will be provided information on how to maintain sleep changes.

**5. Booster sessions**

- In this brief (approximately 20 minutes) telephone session, techniques from sessions 1 to 4 will be reviewed. The therapist moderator will encourage continued practice of techniques and assist in troubleshooting of problems.

**Session content for NiteCAPP SHARES.**

**1. Expanded sleep education and sleep hygiene**

- Participants are provided sleep education regarding sleep and the brain, mood, behavior, health, and weight. Sleep hygiene will be taught and participants are instructed to adhere to the following rules (the goal of sleep hygiene is to eliminate sleep-interfering behaviors):
  avoid caffeine after noon
  within 2 hours of going to bed, avoid exercise and heavy meals
  within 1 hour of bedtime, avoid screen time
  use the bed for sleeping only

**2. Insomnia education and sleep hygiene support**

- Participants are provided education on sleep stages and cycles, sleep disorders, and safety precautions regarding sleep.

**3. Targeted sleep education and sleep in dementia**

- Participants are provided targeted sleep education and education about sleep in dementia.

**4. Review and education and plan for maintenance of behavior change**

- Sleep prescription for caregiver and person with dementia will be updated as appropriate. Caregiver and person with dementia will be provided information on how to maintain sleep changes.

**5. Booster sessions**

- In this brief (approximately 20 minutes) telephone session, techniques from sessions 1 to 4 will be reviewed. The therapist moderator will encourage continued practice of techniques and assist in troubleshooting of problems.

#### Treatment Integrity

The 3-step method formulated by Lichstein [37] will be used to measure treatment integrity.

##### Step 1: Treatment Delivery and Training

Moderators use web-based manuals. Practice begins with mock web-based sessions (ie, moderator and patient interactions), followed by web-based sessions with volunteers. The principal investigator (CSM), a licensed psychologist who is board certified in behavioral sleep medicine, will score all training sessions. Training lasts approximately 8 weeks until the moderators obtain mastery (scoring 100 on each session’s *Moderation Score Sheet*). All moderator-provided feedback will be delivered through email. Transcripts of moderator and participant interactions will be stored, and 25% will be scored by the consultant (a licensed psychologist). A senior consultant (a licensed psychologist) will double score the initial 10 sessions and 10 boosters and then 10% of the remaining sessions for reliability. Consultants will inform the principal investigator (CSM) of scores <95% for supervisory and training purposes. The principal investigator (CSM) will review 25% and the moderators will review 25% of each other’s sessions for ongoing training and supervision. Only consultant reviews will be used to assess fidelity.

##### Step 2: Treatment Receipt

Each week, participants will be emailed a link to their assigned session. Automatic prompts and moderator emails will be used to encourage session completion. To ensure treatment comprehension, participants will be encouraged to ask questions using the embedded communication portal. Moderators will monitor and respond within 24 hours to messages sent through the system. Web-based materials will describe and reinforce treatment content. Time spent viewing web-based sessions will be monitored and recorded. Participants will complete a brief web-based quiz at the end of session 2.

##### Step 3: Treatment Enactment

To ensure that assignments are completed (eg, relaxation session completed and instructions followed), web-based materials contain simple written instructions. Participants will maintain daily electronic diaries and logs. Participants will complete a patient satisfaction and experience survey at the posttreatment and follow-up assessments. Reasons for withdrawal will be assessed using a withdrawal questionnaire.

#### Treatment Credibility and Expectancy

At the end of session 2, participants will complete a treatment credibility questionnaire. The treatment credibility questionnaire is a 4-item scale that assesses the participant’s reaction to therapist and treatment efficacy, and participants provide ratings of 1 (strongly disagree) to 10 (strongly agree). Higher scores represent better treatment credibility.

### Outcomes

A summary of study outcome measures is provided in Table 1 and schedules of outcome and process measures are provided in Tables 2 and 3.

**Table 1:** Outcome measures.

Outcome category and measure			Details
**Aim 1: feasibility and acceptability**			
	**Completion and adherence**		
		Percentage of sessions completed	Percentage of sessions completed
		Five instructions followed on logs	Percentage of instructions followed as indicated on treatment adherence logs
	**Utility and treatment satisfaction**		
		Internet Intervention Utility Questionnaire [38]	This is a 16-item measure designed to assess usability, likeability, usefulness, understandability, and convenience of an internet intervention using a 5-point Likert scale, ranging from 0 (not at all) to 4 (very); 2 additional open-ended questions ask about the most and least helpful aspects of the program.
		Satisfaction Survey	This is a 9-item measure designed to provide feedback on the study, including its structure, assessments, scheduling, working with study staff, and the usability of the intervention platforms using a 10-point Likert scale, ranging from 1 (strongly disagree) to 10 (strongly agree), and open-ended questions.
**Aim 2: primary and mechanistic caregiver outcomes**			
	**Sleep**		
		Daily sleep diaries	Daily electronic diaries assess caregiver sleep onset latency (lights out until sleep onset), wake after sleep onset, and sleep efficiency (total sleep time/time in bed × 100%).
		Insomnia Severity Index [39]	This is a 7-item measure designed to assess the nature, severity, and impact of insomnia using a 5-point Likert scale, ranging from 0 (no problem) to 4 (very severe problem).
	**Arousal**		
		Peripheral arousal: HRV^a^	HRV will be assessed using a Holter monitor during an established stress reactivity protocol. Participants will be seated at rest and undergo Holter monitoring procedures: (1) 5 minutes, baseline; (2) 30 seconds, vibrotactile stimuli (Conair WM200X) at 80 Hz oscillations applied to left hand; (3) 3 minutes, recovery; (4) 30 seconds, vibrotactile stimuli applied to right hand; (5) 3 minutes, recovery; (6) cold pressor stimulation to right hand (place hand at bottom of bowl of ice water calibrated to 4 °C); (7) 3 minutes, recovery; and (8) cold pressor stimulation to left hand. Time and spectral analysis of short-term HRV during baseline, vibration, and cold pressor stimuli will be conducted using Pathfinder software (Spacelabs Healthcare). Time (reflects beat-to-beat variability: RMSSD,^b^ pNN50^c^) and frequency (reflects underlying HR^d^ rhythms: high, 0.15-0.4 Hz; low, 0.04-0.15 Hz; very low, <0.04 Hz, and LF^e^:HF^f^ ratio] domains will be examined. Vibration and cold pressor hand placement order will be counterbalanced every 10 participants to account for order effects.
		Chronic stress: hair cortisol	With permission, research staff will cut hair strands as close as possible to scalp (forearm if needed [40]; minimum 50 mg of hair per sample).
		Perceived Stress Scale [41]	This is a 10-item measure designed to assess past-month stress levels in response to everyday situations using a 4-point Likert scale ranging from 0 (never) to 4 (very often).
		Kingston Caregiver Stress Scale [42]	This is a 10-item measure designed to assess three categories—caregiving, family, and financial issues—using a 5-point Likert scale ranging from 1 (feeling fine or no stress) to 5 (extreme stress).
		Dysfunctional Beliefs and Attitudes about Sleep [43]	This is a 30-item measure designed to assess dysfunctional beliefs and attitudes about sleep using a 10-point Likert scale ranging from 0 (strongly disagree) to 10 (strongly agree).
**Aim 3: secondary health-related caregiver outcomes**			
	**Health and mood**		
		SF-36^g^ [44]	This is a 36-item measure designed to assess quality of life using a 5-point Likert scale ranging from 1 (poor) to 5 (excellent).
		Beck Depression Inventory [45]	This is a 21-item measure designed to assess depressive symptomatology using a 4-point Likert scale ranging from 0 (absence of symptoms) to 3 (severe).
		State-Trait Anxiety Inventory [46]	This is a 20-item measure designed to assess anxiety using a 4-point Likert scale ranging from 1 (not at all) to 4 (very much so).
	**Burden**		
		Zarit Burden Scale [47]	This is a 22-item measure designed to assess burden using a 5-point Likert scale ranging from 0 (never) to 4 (nearly always).
		Quality of life of caregiver of patient with dementia [48]	This is a 20-item measure designed to assess how caregiver quality of life changes after beginning caregiving using yes or no questions and a 10-point sliding scale ranging from 0 (easy) to 10 (hard).
	**Cognition**		
		Daily Joggle battery [49]	Web-based battery designed to assess daily cognitive performance in multiple domains, including processing speed and attention (psychomotor vigilance and digit symbol substitution tasks), visuospatial ability and memory (visual object learning and line orientation tasks), verbal learning and memory (auditory verbal learning task), and executive functioning and working memory (abstract matching and n-back tasks).
		NIH Toolbox for Assessment of Neurological and Behavioral Function [50]	Computerized measure designed to assess cognitive performance in multiple domains, including processing speed and attention (pattern comparison task), visuospatial ability and memory (picture sequence learning task), verbal learning and memory (auditory verbal learning task), and executive functioning and working memory (dimensional card sort and a flanker inhibition tasks).
		Cognitive Failures Questionnaire [51]	This is a 25-item measure designed to assess an individual’s perception of their own daily cognitive failures (eg, absentmindedness and memory errors) using a 5-point Likert scale ranging from 0 (never) to 4 (very often).
	**Inflammation**		
		Blood-based biomarkers	High-sensitivity CRP^h^ and IL-6^i^. Plasma proteins are digested with trypsin, and peptides specific for CRP and IL6 are quantified using multiple reaction monitoring mass spectrometry.
	**Neurodegeneration**		
		Blood-based biomarkers	Aβ^j^40/42, tau, p-tau^k^-181, and p-tau-217. Plasma is extracted (Aβ40/42), or plasma proteins are digested with trypsin, and peptides are quantified using multiple reaction monitoring mass spectrometry.
**Aim 4: persons with dementia: secondary outcome (sleep change in persons with dementia)**			
	**Actigraphy**		Actiwatch 2 (Philips Respironics) is a watch-like device that will be worn 24/7 during each 1-week assessment to monitor light and gross motor activity. Data will be analyzed using 30-second epochs and a validated algorithm (Actiware-Sleep version 3.3; Mini Mitter Co, Inc) to estimate total wake time.
**Exploratory aim: moderators**			
	**Demographics**		
		Demographics	Socioeconomic status, race and ethnicity, education level, marital status, financial strain, residence, menopause symptoms, medications, comorbidities, distance (miles) to the nearest provider, and rurality (Rurality Index [52]).
	**Interpersonal**		
		Patient-Caregiver Functional Unit Scale [53]	This is a 43-item measure designed to assess how much assistance the person with dementia needs and caregiver feelings about helping using yes or no questions and 2 types of Likert scales: a 3-point scale ranging from 0 (without help) to 2 (completely unable) and a 4-point scale ranging from 0 (no) to 3 (both physically and emotionally difficult).
	**Shared lifestyle**		
		Godin-Shephard Leisure-Time Physical Activity Questionnaire [54]	This is a 3-item measure designed to assess how many times per week an individual engages in mild, moderate, or strenuous activity and asks the number of times per week the items were accomplished.
		Fruit and vegetable servings	Amount of fruit and vegetable servings per day
	**Respite and assistance**		
		Respite	Amount and type of respite
		Assistance	Assistance provided by secondary informal caregivers (number, relationship, hours: face-to-face vs other).
	**Dementia severity of persons with dementia**		
		Dementia Severity Rating Scale [55]	This is an 11-item measure designed to assess severity of functional and cognitive decline in patients with Alzheimer disease using various Likert scales.
	**Nighttime behaviors of persons with dementia**		
		Neuropsychology Inventory Nighttime Behavior Scale [56]	This is an 8-item measure designed to assess frequency and severity of detrimental sleep behaviors, such as wandering, in the dementia patient using yes or no questions.

^a^HRV: heart rate variability.

^b^RMSSD: root mean square of successive RR interval differences. RR interval is the time between 2 detected heartbeats, calculated for every QRS event.

^c^pNN50: The proportion of NN50 divided by the total number of NN (R-R) intervals. NN50 is the number of times successive heartbeat intervals exceed 50 ms. RR interval is the time between 2 detected heartbeats, calculated for every QRS event. NN interval is the time (normalized) between 2 detected heartbeats, calculated for every QRS event.

^d^HR: heart rate.

^e^LF: low frequency.

^f^HF: high frequency.

^g^SF-36: Short Form-36 Health Survey Questionnaire.

^h^CRP: C-reactive protein.

^i^IL-6: interleukin-6.

^j^Aβ: plasma amyloid beta.

^k^p-tau: phosphorylated tau.

**Table 2:** Schedule of outcome measures.

	Baseline	Treatment	After treatment	Boosters	6-month follow-up	12-month follow-up
Assessment period, weeks	1	4	1	1	1	1
Screening, apnea testing, consent and assent, and demographics	✓					
HRV,^a^ ISI,^b^ PSS,^c^ KCGSS,^d^ inflammation and neurodegeneration biomarkers, cortisol, actigraphy, SF-36,^e^ BDI,^f^ STAI,^g^ DBS,^h^ ZBS,^i^ QoL,^j^ daily Joggle battery, NIH Toolbox for Assessment of Neurological and Behavioral Function, CFQ,^k^ P-CG FUS,^l^ G-S L-T PAQ,^m^ fruit and vegetable servings per day, respite, secondary CG^n^ outcomes, DSRS,^o^ NPI,^p^ and NBS^q^	✓		✓		✓	✓
Electronic daily diaries	✓	✓	✓	✓	✓	✓

^a^HRV: heart rate variability.

^b^ISI: Insomnia Severity Index.

^c^PSS: Perceived Stress Scale.

^d^KCGSS: Kingston Caregiver Stress Scale.

^e^SF-36: Short Form-36 Health Survey Questionnaire.

^f^BDI: Beck Depression Inventory.

^g^STAI: State-Trait Anxiety Inventory.

^h^DBS: Dysfunctional Beliefs about Sleep.

^i^ZBS: Zarit Burden Scale.

^j^QoL: Quality of life of caregiver of patient with dementia.

^k^CFQ: Cognitive Failures Questionnaire.

^l^P-CG FUS: Patient-Caregiver Functional Unit Scale.

^m^G-S L-T PAQ: Godin-Shephard Leisure-Time Physical Activity Questionnaire.

^n^CG: caregiver.

^o^DSRS: Dementia Severity Rating Scale.

^p^NPI: Neuropsychological Inventory.

^q^NBS: Nighttime Behavior Scale.

**Table 3:** Schedule of process measures.

	Baseline	Treatment	After treatment	Boosters	6-month follow-up	12-month follow-up
Assessment period (weeks)	1	4	1	1	1	1
Treatment integrity: delivery and receipt and enactment		✓		✓		
Treatment credibility: quiz and improvement expectancy		✓	✓		✓	✓
Sleep knowledge and working alliance		✓	✓	✓	✓	✓

### Study Timeline

The study timeline is provided in Table 4.

**Table 4:** Study timeline.

	Project year										
	1		2		3		4			5	
	First half	Second half	First half	Second half	First half	Second half	First half	Second half	First half		Second half
Develop manual of operating procedures, register with ClinicalTrials.gov, publish trial protocol, and train moderators and assessors	✓										
Recruit, collect baseline measurements, and deliver treatment		✓	✓	✓	✓	✓	✓				
Collect assessment after treatment		✓	✓	✓	✓	✓	✓	✓			
Collect 6- and 12-month follow-up assessments			✓	✓	✓	✓	✓	✓	✓		
Offer and provide NiteCAPP CARES^a^ to NiteCAPP SHARES^b^ participants				✓	✓	✓	✓	✓	✓		✓
Final data analysis and dissemination (continues after grant ends) and final report									✓		✓

^a^NiteCAPP CARES: cognitive behavioral therapy for insomnia.

^b^NiteCAPP SHARES: active web-based sleep hygiene and related education control.

### Analytical Approach

#### Power Analysis

Power will be simulated using SAS 9.4 statistical software (SAS Institute Inc) using a generalized estimating equations (GEE) design (clusters are individuals measured over 4 time points) with an autoregressive working correlation structure (correlation decreases as time point distance increases, *ρ*=0.5, 1 million Monte Carlo integrations simulated) [57]. The approach described by Schluchter [58] will fit GEE models with and without mediator to estimate mediated effect (exploratory aim). Sample size of 80 is powered (>.80) to detect small to large Cohen *d* mediation effects. Given an anticipated dropout rate of 15% to 20%, we will recruit a total sample of 120.

#### Evaluations of Aims

##### Tests of Hypotheses

All analyses will use intent-to-treat using all randomized participants. All assumptions will be tested. If the normality assumption is violated, the nonparametric Mann-Whitney *U* test will be used.

##### Testing of Aim 1: Feasibility and Acceptability of NiteCAPP CARES and NiteCAPP SHARES

The feasibility and acceptability of NiteCAPP CARES and NiteCAPP SHARES will be evaluated using average completion rates (number of sessions completed), adherence (measured through sleep diaries and logs), and satisfaction and utility ratings. These variables will be compared for NiteCAPP CARES and NiteCAPP SHARES using independent samples 2-tailed *t* tests.

##### Testing of Aims 2 to 4: Primary and Mechanistic and Secondary Outcomes

**Figure 3:**
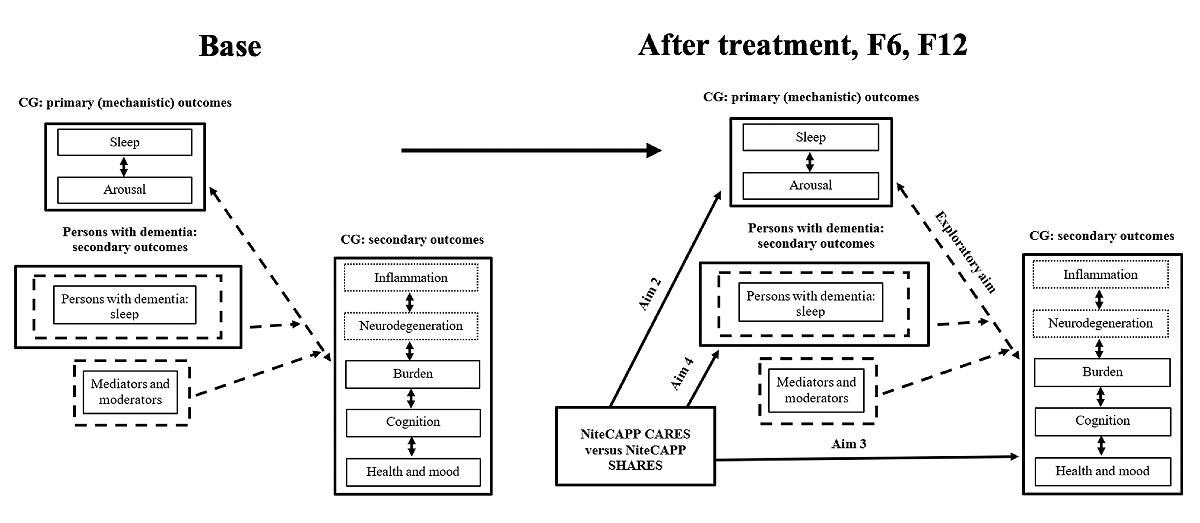
Mediated generalized estimating equations model. Analyses will also be conducted examining the paths from after treatment to 6-month follow-up and from 6-month follow-up to 12-month follow-up. Base: baseline; CG: caregiver; F6: 6-month follow-up; F12: 12-month follow-up; NiteCAPP CARES: cognitive behavioral therapy for insomnia; NiteCAPP SHARES: active web-based sleep hygiene and related education control; Post: after treatment.

A mediated GEE model [58] (clusters are individuals measured over 4 time points) will test causal paths between intervention effects on the primary and secondary outcomes (Figure 3). On the basis of a priori hypotheses, separate GEE models, including group (NiteCAPP CARES and NiteCAPP SHARES), time (baseline, after treatment, and 6-month and 12-month follow-ups), and their interaction as predictors, will be conducted for each primary CG outcome (aim 2: insomnia severity, sleep onset latency, wake after sleep onset, sleep efficiency, arousal, and inflammation), secondary CG outcome (aim 3: health, mood, burden, and cognitive performance), and secondary outcome for persons with dementia (aim 4: actigraphy total wake time for persons with dementia). Potential covariates affecting the mechanistic outcomes (Table 2) or technology use by CGs and persons with dementia (eg, Internet Intervention Utility Questionnaire [38]) will be assessed and used as covariates if significant.

##### Testing of Exploratory Aim

We will evaluate primary and secondary outcome change relationships and their potential mediators and moderators (Table 2). Mediated GEE will be used to compare NiteCAPP CARES versus NiteCAPP SHARES. Moderating effects will be tested using interactions (Table 2).

### Missing Values

The SAS PROC GEE weighted model handles missing data completely at random or at random. When measures are collected over time, some participants may not complete the study. Unlike repeated measures ANOVA, which excludes them from analysis, weighted GEE retains them (increasing power and producing unbiased estimates).

### Patient and Public Involvement

Patients and the public are not involved in any of the following study procedures: development of research questions and outcome measures or plan for results dissemination. The Community Advisory Board (CAB) will assess the burden of the intervention and may help with recruitment. Every participant will fill out a patient satisfaction and experience survey after treatment and at 6-month and 12-month follow-ups. This questionnaire asks CGs and persons with dementia which modules were most and least useful for them, how they felt about the length of the program, suitability for CGs and persons with dementia, and so on. Trial results will be communicated to participants and the public through peer-reviewed manuscripts.

### Ethics Approval

All procedures were approved by the University of Missouri Institutional Review Board on May 6, 2021 (2053682); safety officer Dr Susan McCurry on November 24, 2021; the National Institute of Aging (NIA) on January 26, 2022; and the University of South Florida Institutional Review Board on March 10, 2022 (003936). The NIA and the safety officer reviewed and approved the study protocol, manual of operating procedures, informed consent form, and monitoring plan with emphasis on data integrity and patient safety issues in November 2021. The safety officer will review these biannually. Any changes to these procedures that are recommended by the safety officer will be adopted with institutional review board and NIA approval. The safety officer will review adverse events and monitor study results, focusing on efficacy, recruitment progress, randomization, compliance, retention, protocol adherence, operating procedures, forms completion, intervention effects, participant safety, and minority inclusion. The principal investigator (CSM) registered the study with ClinicalTrials.gov (NCT04896775) on May 21, 2021, and will submit annual reports to the funding agency.

Our team includes a CAB comprising 8 CGs, 4 persons with dementia, and 4 local experts. The CAB structure and procedures are based on the CAB Toolkit described by Kubicek and Robles [59]. The CAB will meet twice each year, with each meeting lasting 2 hours (respite care costs for persons with dementia will be paid by the grant). The team includes a consultant CG advocate who is a trained facilitator, and she will facilitate the CAB meetings.

## Results

This work is supported by the National Institutes of Health’s NIA (R01AG066081). Recruitment procedures started in February 2022. All data are expected to be collected by 2026. Full trial results are planned to be published by 2027. Secondary analyses of baseline data will be subsequently published.

We will present the findings at national conferences, including the Associated Professional Sleep Societies (APSS or SLEEP) and the Gerontological Society of America, in the final year of the project. An abstract reporting the findings of the preproposal focus group was presented at the August 2020 internet-based SLEEP meeting, and a brief report based on these findings is currently under review. The web-based treatment materials will be shared electronically and will be widely available to clinicians. The findings will also be disseminated to the dementia and dementia caregiving communities through websites and other resources. The team’s community CG advocate and CAB will be involved in planning for broad dissemination to the dementia and CG communities. They will also help to ensure that materials used to disseminate findings are written for, and easily understood by, lay audiences in rural areas.

## Discussion

The overarching goal of this RCT is to evaluate the acceptability, feasibility, and short-term and long-term effects of NiteCAPP CARES on the sleep and stress mechanisms underlying poor CG health and functioning. To the best of our knowledge, there is no current web-based CBT-I treatment that is tailored for CGs of persons with dementia and none that involve the person with dementia in treatment.

### Strengths and Limitations

This study includes several strengths. It is a novel 4-week web-based CBT-I (NiteCAPP CARES) in CGs and persons with dementia (using a dyadic approach) that integrates sleep education, sleep hygiene, stimulus control, sleep compression, relaxation, problem solving, coping and stress management, and cognitive restructuring techniques. This study uses a rigorous methodology and NiteCAPP CARES will be examined in comparison with an active web control (NiteCAPP SHARES). Long-term follow-ups at 6 and 12 months will enable examination of the persistence of the behavioral outcomes of NiteCAPP CARES. The potential limitations include participant attrition at follow-up, which may contribute to selection bias associated with systematic differences between participants completing NiteCAPP CARES and those completing NiteCAPP SHARES.

### Future Directions

Future directions for this RCT include a multisite effectiveness trial to determine generalizability across different areas. In addition, once NiteCAPP CARES is enhanced based on the results of this RCT, it needs to be tested within other populations (nonrural CGs and other underserved populations) for broader dissemination. Finally, dismantling studies could examine the most effective components of NiteCAPP CARES treatment to further streamline the treatment.

### Conclusions

This RCT tests NiteCAPP CARES, a web-based CBT-I for rural CGs. The Cognitive Activation Theory of Stress and our research as well as research conducted by others [7,31] support our novel hypothesis that NiteCAPP CARES will improve CG health, mood, burden, and cognition by targeting sleep, arousal, and inflammation. This trial addresses what improves as well as how, why, and for how long (up to 1 year). This trial is a necessary first step, and the results from this study will help us to modify NiteCAPP CARES to optimize treatment potency and support future pragmatic testing and dissemination.

## Appendix

Multimedia Appendix 1 Peer-reviewer report from the Center for Scientific Review Special Emphasis Panel Member Conflict: Stress, Sleep, Disparities, and Aging (National Institutes of Health, USA).

## Abbreviations

ANS: autonomic nervous system
Aβ: plasma amyloid beta
bCBT-I: brief cognitive behavioral therapy for insomnia
CAB: Community Advisory Board
CBT-I: cognitive behavioral therapy for insomnia
CG: caregiver
GEE: generalized estimating equations
HR: heart rate
HRV: heart rate variability
NiteCAPP CARES: cognitive behavioral therapy for insomnia
NiteCAPP SHARES: active web-based sleep hygiene and related education control
RCT: randomized controlled trial

## Disclaimer

The study sponsor was not actively responsible or involved in the study design and will have no involvement in collection, management, analysis, or interpretation of data. The sponsor will have no involvement in future manuscript preparation and decision to submit for publication. This research was conducted using previous patient involvement in the pilot study. Patients were invited to comment on the feasibility and acceptability design of NiteCAPP CARES, and they indicated that more simple language would be preferable. In response, for this randomized controlled trial, we edited NiteCAPP CARES to simplify the language. Patients were invited to contribute to the writing and editing of this document for readability or accuracy.
