# Supplementary material for: Impact of Web-Based Cognitive Behavioral Therapy for Insomnia on Stress, Health, Mood, Cognitive, Inflammatory, and Neurodegenerative Outcomes in Rural Dementia Caregivers: Protocol for the NiteCAPP CARES and NiteCAPP SHARES Randomized Controlled Trial
Source: JMIR Res Protoc. 2022 Jun 14;11(6):e37874. doi: 10.2196/37874 (PMC9240954; doi:10.2196/37874)
Supplement: Multimedia Appendix 1 [file resprot_v11i6e37874_app1.pdf]

SUMMARY STATEMENT

PROGRAM CONTACT: (Privileged Communication) *Release Date:* 11/24/2020

Miroslaw Mackiewicz
301-496-9350
mackiewiczm2@mail.nih.gov

Revised Date:

Application Number: 1 R01 AG066081-01A1
Formerly: 1R01AG066081-01A1

Principal Investigator

MCCRAE, CHRISTINA S

Applicant Organization: UNIVERSITY OF MISSOURI-COLUMBIA

Review Group: ZRG1 BBBP-S (02)
Center for Scientific Review Special Emphasis Panel
Member Conflict: Stress, Sleep, Disparities, and Aging

Meeting Date: 11/16/2020 *RFA/PA:* PA20-183
Council: JAN 2021 *PCC:* 3BBSDMA
Requested Start: 01/01/2021

Project Title: Web-based CBT for Insomnia in Rural Dementia Caregivers: Examination of Sleep, Arousal, Mood, Cognitive, and Immune Outcomes
SRG Action: Impact Score:31 Percentile:16 #
Next Steps: Visit https://grants.nih.gov/grants/next_steps.htm
Human Subjects: 30-Human subjects involved - Certified, no SRG concerns
Animal Subjects: 10-No live vertebrate animals involved for competing appl.
Gender: 1A-Both genders, scientifically acceptable
Minority: 1A-Minorities and non-minorities, scientifically acceptable
Age: 3A-No children included, scientifically acceptable

Project Year	Direct Costs Requested	Estimated Total Cost
1	495,130	742,666
2	493,776	740,635
3	490,081	735,093
4	490,838	736,229
5	488,533	732,771
TOTAL	2,458,358	3,687,395

ADMINISTRATIVE BUDGET NOTE: The budget shown is the requested budget and has not been adjusted to reflect any recommendations made by reviewers. If an award is planned, the costs will be calculated by Institute grants management staff based on the recommendations outlined below in the COMMITTEE BUDGET RECOMMENDATIONS section.
BUDGET MODIFICATIONS

1R01AG066081-01A1 Mccrae, Christina

COMMITTEE BUDGET RECOMMENDATIONS

RESUME AND SUMMARY OF DISCUSSION: This resubmitted application proposes to conduct an initial controlled efficacy trial of a web-based cognitive behavioral therapy intervention for insomnia (CBT-I), among family caregivers of dementia, and assess biopsychosocial markers of change. This project has the potential impact of providing the field with a low-cost, disseminable, tailored intervention to treat a vulnerable group of individuals in a rural setting. The investigators were highly responsive to prior critiques by further developing and pilot testing the intervention, providing clarification of the expertise of the team, broadening enrollment, modifying the analytic plan, and including a DSMB, among several others. The investigative team is excellent, has extensive expertise in clinical trials for insomnia, research with older adult, and biostatistics, and has demonstrated a track record of productive collaboration; however, there was a relative limitation in the expertise in psychophysiology and neuroendocrinology represented on the team. The environment is similarly excellent and has the necessary facilities, equipment, and recruitment resources, to carry out this proposed clinical trial. The use of a tailored web-based CBT-I protocol for this population and the approach to examining CG and PWD dyads, were considered innovative aspects of this proposal, even though many of the individual aspects of been studied prior. The reviewers identified several strengths in the approach including the supportive preliminary data, the inclusion of the community advisory board, feasible recruitment strategy, the remote intervention and assessment strategies, and excellent attention to the rigor and reproducibility associated with clinical trial methodology. The panel also discussed several limitations that remained which primarily centered on the biological aspects of the proposal. The conceptual model for measuring the neuroendocrine and psychophysiological pathways lacked clarity and appeared circular. In line, there was a lack of preliminary support for these proposed measures, the hypothesized directions of the effects of the intervention were unclear, and several aspects of the proposed measurements for these markers were insufficient that reduced the rigor and reproducibility of the outcomes of these aspects of the proposal. However, the panel concluded that the aforementioned limitations only modestly reduced the likelihood of this project having a sustained and powerful influence on the field of sleep medicine.

DESCRIPTION (provided by applicant): Compared to their urban counterparts, rural family dementia caregivers (CGs) face increased vulnerability to insomnia and related health concerns (stress, inflammation, depression, anxiety, cognitive disturbance). Cognitive behavioral treatment for insomnia (CBT-I) holds promise for improving insomnia and these related concerns, but is difficult to access in rural areas. Our team developed brief telehealth CBT-I (tele bCBT-I) tailored for CGs (e.g., includes stress management/problem solving) that improved sleep, arousal, mood, cognition and inflammation (small to large effects). While telehealth improves accessibility, it is still burdensome for CGs due to inflexible scheduling and scarcity of trained therapists. Thus, more research is needed. Web delivery would increase access and web CBT-I is efficacious in non-CG adults, but has not been tested in rural CGs. Using the NIH Stage Model flexible framework and Medical Research Council recommendations, we developed NiteCAPP (web translation of our tele bCBT-I protocol). Stage IA/B validation and testing show high feasibility and acceptability, and improvements in sleep, arousal, mood, burden and cognition in a single arm pilot in rural CGs (n=5). The proposed trial is the next logical step - Stage II testing in an RCT (n=100) to establish efficacy and further evaluate feasibility and acceptability. The Cognitive Activation Theory of Stress provides a framework for our basic premise that CGs experience insomnia, arousal and inflammation that prompt sympathetic activation and hypothalamic-pituitary-adrenal (HPA) disruption that have downstream negative effects on health. The proposed trial tests the novel hypothesis that NiteCAPP will improve CG health, mood, burden and cognition by targeting their shared underlying mechanisms – sleep, arousal and inflammation – thereby, returning sympathetic and HPA functioning to normal. Another novel aspect of the proposed trial is inclusion of behavioral strategies to target the person with dementia’s (PWD) sleep. Outcomes will be assessed at baseline,

post-treatment and two follow-ups (6 and 12 months) and include CG sleep, arousal, inflammation, health, mood, burden and cognition, and PWD sleep. The proposed study has four specific aims. Aim 1 focuses on the feasibility and acceptability of NiteCAPP and WebSHE (sleep hygiene education – active web comparator). Aims 2 and 3 examine NiteCAPP's effects (versus WebSHE) on CG primary/mechanistic (sleep, arousal, inflammation) and secondary outcomes (health, mood, burden, cognition), respectively. Because the PWD's sleep impacts CG sleep, Aim 4 examines NiteCAPP's secondary effects on PWD sleep (objectively assessed). An Exploratory Aim examines the relationships between changes in CG primary and secondary outcomes, and their potential mediators/ moderators. Public Health Implications: Demonstration that rural CGs can use NiteCAPP to target sleep, arousal/stress, inflammation and related health concerns has important implications for multiple stakeholders, including rural CGs, rural PWDs, their families, clinicians and policymakers.

PUBLIC HEALTH RELEVANCE: This trial uses internet technology and community methods to test the acceptability, feasibility and efficacy of NiteCAPP, a brief web CBT-I for rural dementia caregivers (CGs). It offers new understanding of how NiteCAPP improves CG sleep, arousal and inflammation and prompts downstream effects on other outcomes (health, mood, burden, cognition). Demonstration that rural CGs can use NiteCAPP to target sleep, arousal, inflammation and related concerns has important implications for rural CGs/PWDs, their families, clinicians and policymakers.

CRITIQUE 1

Significance: 3
Investigator(s): 1
Innovation: 4
Approach: 2
Environment: 2

Overall Impact: This is a resubmission of an Investigator-initiated R01 that proposes to develop a Web-based Cognitive Behavior Therapy program for Insomnia (CBT-I) for caregivers (CGs) of Persons With Dementia (PWD) living in rural environments. The Principal Investigator is outstanding, the Co-Investigators strong and the Environment is excellent. The Resubmission is extraordinarily responsive to the prior Summary Statement in making numerous changes and modifications to the application, perhaps the most substantive being the development and pilot testing of the Web-based package, as well as the inclusion of both genders as CGs, the inclusion of a conceptual model (Cognitive Activation Theory of Stress), a fully developed data analytic plan including Generalized Estimating Equations, elucidation of a Data Safety Monitoring Board, a rationale to study CGs in rural environments, and inclusion of Co-Investigators to enhance recruitment. An additional asset of the application is its alignment with the NIH Science of Behavioral Change initiative. The usage of actigraph data derived from the PWD constitutes a potentially important aspect of understanding mediational effects. Despite these many strengths, there remain some issues regarding the selection of plasma markers, and the particular Heart Rate Variability measure selected here is not well-justified, though it could still serve adequately in the mediational models. The Web-based implementation of CBT-I with rural CGs of PWD can be construed as nominal repackaging of well-established behavioral approaches for sleep interventions that have already been applied by the Principal Investigator and others to many other patient groups, including some in rural setting, albeit in a different venue. The degree of novelty is thus incremental, rather than fundamental, and this impacts the whole of the application, which is generally well-done but not exceptionally innovative.

1. Significance

Strengths

- Caregivers (CGs) of persons with dementia (PWD) undergo enormous amounts of stress in their roles as taking care of such individuals, and those stressors have widespread and substantial impact on psychological and medical burden; treatments, particularly ones that involve behavioral approaches to alleviate some of those stressors by improving sleep, represent an important topic of research
- Application appears heavily aligned with goals of the trans-institute NIH Science of Behavioral Change initiative

Weaknesses

- A purely CBT approach to factors impacting sleep disruption may miss some of the more salient psychodynamic issues impacting CGs as the they experience the “slow death of mind” of the PWD; this seemed to be implied in at least one on-line program (not focused on sleep issues) in CGs (ref 25)

2. Investigator(s)

Strengths

- The Principal Investigator (McCrae) is a top-notch behavioral health/clinical psychologist with both considerable academic and clinical experience; she is superbly suited to conduct the proposed work; she is considered one of the foremost authorities on Cognitive-Behavior Therapy for Insomnia (CBT-I) across a wide range of populations (including the elderly) and has a proven track record of publications in that area
- Previous review contained comments minimizing the Principal Investigator’s background in caregiver research, the basis of which appear unfounded in the PI’s publications, which includes 4 papers specifically dealing with caregivers’ sleep issues between 2008 and 2018
- The Principal Investigator’s association with Dr. Rowe (USF) has been productive and longstanding
- Other colleagues are strong

Weaknesses

- None obvious

3. Innovation

Strengths

- The dyadic model examining the PWD’s sleep behavior as a mediator of improvement in CG sleep is innovative
- The development of a Web-based program targeting poor sleep in rural caregivers of PWD is a slightly different extension of CBT-I using a different modality of presentation to an under-resourced population

Weaknesses

- Lack of innovation of the particular work described here is based on the following facts: a) web-based programs for poor sleep using CBT principles are not novel in groups other than CGs (Table 4 on pg. 118); b) studies of CBT-I in CGs are not novel (refs 44; 45); c) CBT-I usage in rural non CG-populations is not novel (e.g., the PI’s own work [RESTORE study; ref 37]); d) Web-based interventions for other health outcomes in CG are not novel.

4. Approach

Strengths

- In response to issues raised on previous review, intervention (NiteCAPP) has been pilot tested and has been the subject of focus groups; these results now well-presented in the application; appropriate care for development of issues specific to older CGs (readability, ease of use, etc.) has been taken; preliminary data from intervention yields > 10% improvements in subjective sleep efficiency, 7-point reduction in ISI and 5-point reduction in BDI, all meaningful and non-trivial effects
- The Principal Investigator is well-connected with the local community of PWD and their CGs and has many letters of interest and support from appropriate administrators documenting this that are included in the application
- The study is grounded in conceptual model (Cognitive Activation Theory of Stress)
- Study will probe mechanism by examining mediators and moderators as predictors of improved sleep (emphasizing not only the “what” but also the “how” and “why”)
- The PI has worked with CBT-I in older rural populations previously (RESTORE studies)
- Web-based delivery of CBT-I makes sense given widespread internet access throughout Missouri
- Appropriate statistical analyses including GEE accounting for missing data, correction for family-wise error, and examination of mediation effects at each successive time point are proposed
- Training plans for therapist/moderators are detailed and specific; plans for monitoring treatment integrity are very explicit and clear

Weaknesses

- Arguably, rurality per se may not be a major an issue in sleep problems, when compared to other factors impacting potential health care disparities, such as race, ethnicity, education or occupation; however, this may not be the case for other components of behavioral health (e.g., obesity), and the Investigators imply that other issues (e.g., stigma) may be more relevant in rural areas
- Rationale for these particular inflammatory markers (IL-6, CRP) is not developed
- Some aspects of the rationale for reducing inflammation, although theoretically conceivable, are somewhat conjectural and impossible to test, e.g., the case for reducing inflammatory markers such as CRP and IL-6 in the CGs as reducing risk for later neurodegenerative disease; by way of example, the size of such effects might be expected to be much lower than CG’s genetic load
- No compelling rationale is offered for exclusive use of time-domain (c.f., frequency domain) HRV measures as the most suitable mediator of the impact of the intervention
- Table 8 (under Aim 2) lists blood-based Alzheimer’s biomarkers (beta amyloid and tau) to be obtained in CG; these are also mentioned by Dr. Mooney (Associate Director of Proteomics Research Core) in his support letter; such biomarkers are typically derived from cerebrospinal fluid; valid plasma-based markers remain under development at this time (Palmqvist et al; JAMA 2020: 324: 772-82; Palmqvist et al, JAMA Neurology 2019: 76: 1060-9); further details were not forthcoming in the application

- More details regarding how specific actigraphic data collected in PWD are to be incorporated into the mediational analyses could be provided

5. Environment

Strengths

- The application contains letters of support from colleagues listed within the University Missouri system (Biostats, Nursing, Neurology, Psychiatry, Family Medicine, and Informatics), from various Consultants outside of University of Missouri, local Alzheimer's Association representatives. This is an impressive show of support.
- Although this is Web-based research, the reputation and physical location of the University of Missouri in Columbia is well-situated to allow access to a statewide population that has a large rural component
- Creation of a Community Advisory Board is an important feature of the proposed work and suggests good integration of services within the broader community of caregivers and other stakeholders

Weaknesses

- None obvious

Study Timeline

Strengths

- A detailed Timeline (providing both Year and Quarter) along with specific tasks and personnel involved in each is provided

Weaknesses

- None; the Timeline is exceptionally thorough

Protections for Human Subjects

Acceptable Risks and/or Adequate Protections

- A thoughtful and well-reasoned discussion of this generally low risk trial is provided.

Data and Safety Monitoring Plan (Applicable for Clinical Trials Only):

Acceptable

- Plans for creation of 4-member DSMB are discussed.

Inclusion Plans

- Sex/Gender: Distribution justified scientifically
- Race/Ethnicity: Distribution justified scientifically
- For NIH-Defined Phase III trials, Plans for valid design and analysis: Not applicable
- Inclusion/Exclusion Based on Age: Distribution justified scientifically
- On original submission of the application, reviewers disagreed on whether it was appropriate to include female PWDs with male caregivers; the Resubmission will allow both male and female

PWDs and female and male caregivers; Projected Enrollment table reflects the fact that most PWDs are likely to be men and CGs are likely to be women; this is scientifically acceptable.

Vertebrate Animals

Not Applicable (No Vertebrate Animals)

Biohazards

Not Applicable (No Biohazards)

Resubmission

- This is resubmission of an R01 originally reviewed by the MESH IRG in June 2019; the Investigators have been extraordinarily responsive to a large number of comments offered by prior Reviewers; their single-page Introduction to this resubmission is a densely packed read that adequately addresses prior IRG comments regarding the original submission

Resource Sharing Plans

Acceptable

Budget and Period of Support

Recommend as Requested

CRITIQUE 2

Significance: 1
Investigator(s): 2
Innovation: 1
Approach: 3
Environment: 2

Overall Impact: This re-submission has been responsive to reviewers criticisms, is responsive to the NIH special interest announcement and proposes a very innovative yet pragmatic approach to helping caregivers (CG) working with patients with dementia (PWD) to manage the debilitating disruption that the PWD sleep disturbance can cause. CGs themselves are a vulnerable population, and are a target group for support and early intervention, to help them maintain their own cognitive functioning and wellbeing, manage their own insomnia and sleep disturbance, while still caring for the loved one with dementia. This proposal takes a train-the-trainer approach to give CGs tools to use for managing sleep disturbance in the PWD, and to provide self-help. It has the potential to transform dementia care in rural settings, and beyond.

1. Significance

Strengths

- Caregivers (CG) of persons with dementia (PWD) are under a great deal of stress as they care for loved ones. They have a rate of risk for dementia that is increased compared with the non-caregiver population. In rural settings, there is little possibility to provide clinician access and even telehealth is challenging to implement. However, internet is readily available in the

community/home. An app supported by a care delivery program that could be used to help support CGs and their PWD partner is highly innovative and pragmatic. It could help transform quality of care in the rural setting where access to medical and dementia support resources is a great challenge. As sleep is one of the most challenging PWD symptoms to manage from a CG perspective, and leads to deterioration of the CG's ability to provide care, the strategies being tested in this proposal have the capacity to make a large social and medical impact, and provide large healthcare savings, as well.

Weaknesses

- No significant weaknesses.

2. Investigator(s)

Strengths

- McCrae has a developing leadership track-record in this area.
- The group has done a first rate job pulling together expertise to vet and develop this innovative approach.

Weaknesses

- Biostatistics and modeling effort could be higher in order to maximize data analysis, integration and explore scaling opportunities.

3. Innovation

Strengths

- While CBTi apps are no longer novel, this proposal is highly innovative in its approach to the CG-PWD dyad.
- The teaching of the CG to help themselves and the PWD, is highly innovative and may be transformative in this realm.
- Weaknesses
- Plenty of challenges but no significant weaknesses.

Weaknesses

- None noted by reviewer.

4. Approach

Strengths

- This is a carefully thought through and planned train the trainer approach to CG support around sleep.
- The use of the community advisory board is exemplary. They have even provided infrastructure and support for respite care costs for PWDs.
- In-home apnea screen.
- The use of an active control is a strength.
- The 24-hour return contact is an asset and will help to ensure success.

- The boost and other retention strategies are innovative and well planned.
- Responsive to the NIH notice of special interest.
- Although not central to the importance of outcome value, the addition of inflammatory mediators (hCRP, IL-6) and autonomic measures (HRV) will potentially demonstrate broader long-term health efficacy.

Weaknesses

- Plans for physiological measures are insufficiently detailed. One might argue that the aim to test the intervention, coupled with evaluation of sleep-wake patterns and self-report indices are significant without the physiological measures, which are underdeveloped with respect to methodologies used.
- Data analysis could be more detailed, but important factors such as handling of missing data are incorporated.

5. Environment

Strengths

- Appropriate.
- Access to rural areas and informatics/computer development support are in place

Weaknesses

- No significant weaknesses.

Study Timeline

Strengths

- The study timeline is appropriate for the study which plans to include 100 dyads.

Weaknesses

- More time for data analysis would be advantageous, but exploratory analyses undoubtedly will continue past the award.

Protections for Human Subjects

Acceptable Risks and/or Adequate Protections

- Maybe it's an error and wasn't deleted from a previous version, but the investigators write, "Likewise, participants whose imaging scans reveal abnormalities will be referred to a neurologist or their primary care physician for follow-up". Is a scan even being conducted?

Data and Safety Monitoring Plan (Applicable for Clinical Trials Only):

Acceptable

- DSMB is planned.

Inclusion Plans

- Sex/Gender: Distribution justified scientifically
- Race/Ethnicity: Distribution justified scientifically

- For NIH-Defined Phase III trials, Plans for valid design and analysis: Scientifically acceptable
- Inclusion/Exclusion Based on Age: Distribution justified scientifically
- The CG is more often a woman and this is built into the rationale for imbalance in subjects. Adult CG is appropriate and justified.

Vertebrate Animals

Not Applicable (No Vertebrate Animals)

Biohazards

Not Applicable (No Biohazards)

Resubmission

- Appropriate response to reviewer concerns.

Resource Sharing Plans

Acceptable

Budget and Period of Support

Recommend as Requested

CRITIQUE 3

Significance: 4
Investigator(s): 2
Innovation: 1
Approach: 4
Environment: 1

Overall Impact: This project is a resubmission of a grant to conduct an initial controlled efficacy trial of NiteCAPP, a web-based CBT for insomnia intervention designed for primary caregivers of individuals with dementia who live in rural settings. Considerable preliminary work has been conducted in support of this adaptation of CBTi for the caregiving population and an initial pilot study provides support for feasibility and acceptability of the approach. The current Stage II trail is designed to provide further support for the feasibility and acceptability of the approach and initial efficacy data. The team plans to recruit 100 CGs and assign them to NiteCAPP or a well-matched, web-based control condition. Both interventions involve 4 x 45 minutes online sessions and 4 booster sessions over a 12-month period. Outcomes will be assessed pre- and post-intervention, and at 6 - and 12-month follow-up. Primary outcomes include sleep parameters, heart rate variability, and blood markers of neurodegeneration and systemic inflammation. Secondary outcomes are CG health, mood, burden, executive function, and the person with dementia’s sleep. There are many strengths of this application. It addresses a significant public health disparity, represents a logical next step in Dr. Mccrae’s research program, and involves an experienced study team that is geographically well placed to focus on health disparities among rural caregivers. In addition, the development of the intervention was well considered and included community input at all stages of the process, and the preliminary data generally supports the approach. The team were also responsive to many of the issues raised on initial review. Weaknesses of the

application focus on the presented conceptual model, in particularly the consideration of proposed physiological mediators ("arousal" and blood markers of neurodegeneration and inflammation) of health/mood, burden and cognitive outcomes. This model and the rationale for focusing on these physiological parameters as primary outcomes remains unclear.

1. Significance

Strengths

- The health of individuals who are caring for loved ones with dementia is a growing public health concern. 63% of caregivers have chronic and enduring insomnia.
- Rural caregivers are at particularly high risk due to difficulty accessing health care.
- A strength of the proposal is the examination of a brief, easily accessible, web-based intervention refined to address the needs of rural caregivers, a group at heightened risk for mental and physical health disparities.
- The use of remote technology to access a rural population.
- Preliminary pilot data supporting feasibility and acceptability of the approach.

Weaknesses

- The Cognitive Activation Theory of Stress model added in the revision is not clear, particularly in regard to the role of physiological pathways. The presented model suggests that "insomnia, arousal and inflammation" prompt CNS changes resulting in adverse health outcomes. Specifically, it is suggested that these factors lead to changes in the sympathetic division of the autonomic nervous system and the HPA axis that contribute to lower health-related QOL, depression, burden and poorer cognitive function. This model lacks clarity. If arousal is defined as activation of the ANS, then it is proposed that arousal leads to arousal? Activation of the sympathetic nervous system and the HPA axis are known to contribute to the control of peripheral inflammation, raising questions about the direction of expected effects. It is proposed that NiteCAPP will decrease peripheral markers of inflammation and thus restore HPA and ANS function. However, an alternate model, supported by existing literature, suggests that sleep disruption dysregulates peripheral pathways (ANS, HPA) and thus results in increased systemic markers of inflammation. In sum, the inclusion of "arousal" and inflammation as primary outcomes is not clearly considered.
- Arousal is assessed by measuring HRV, which provides a better measure of activation of the parasympathetic division of the ANS, than the sympathetic division. If the primary mechanisms of interest are the SNS and HPA axis, then better measures are available.
- Arousal is not clearly defined and references to "improved physiological arousal" and "impaired ANS function" are unclear.
- Preliminary work provides considerable support for the proposed sleep outcomes. However, preliminary evidence in support of the inclusion of markers of inflammation/ neurodegeneration is weak or missing.
- No rationale is provided for the proposed peripheral markers of neurodegeneration.
- The Authors are currently conducting a second pilot trial. It is not clear how this trial will inform the proposed work, particularly if it does not support/replicate evidence for proposed pathways.

2. Investigator(s)

Strengths

- Dr Mccrae is an experienced practitioner with an impressive history of running clinical trials to examine the efficacy of CBTi.
- The study team is impressive and covers much of the expertise necessary to conduct the proposed trial.

Weaknesses

- The team would benefit from expertise in psychophysiology/neuroendocrinology given the proposed pathways. Although Co-I Curtis is reported to have taken a course in HRV, she does not mention this in her letter and there are concerns about how this measure is understood in the proposal.

3. Innovation

Strengths

- The proposal is seen as innovative. Particularly, the development of a user-friendly intervention designed to address insomnia and stress among rural caregivers with limited access to other treatment options.
- The adaptation of traditional CBTi to address other issues confronted by caregivers, e.g., stress.
- Adaptation of the intervention to target sleep in the PWD
- Involvement of CG peer advocate.
- Use of booster sessions and plan to follow participants for 12-months.

Weaknesses

- The sections on autonomic arousal and inflammation are not well developed.

4. Approach

Strengths

- The proposed trial is well considered and an appropriate next step for the phase of intervention development.
- Recruitment of an appropriate sample of caregivers with insomnia is carefully considered and appears feasible.
- The intervention has been carefully developed and well adapted to meet the needs of the target population, with engagement of the community at all stages of refinement of the approach.
- Adaptation of traditional CBTi to include challenges affecting rural caregivers, e.g., the addition of problem solving and stress management.
- The inclusion of the PWD in the intervention either directly or through strategies given to the caregiver.
- The study design appears appropriate for the phase of intervention development, including the use of a matched, active control condition.
- Integrity of the intervention is carefully considered.
- The engagement and participation of a community board to help direct the project is a clear strength.

- The remote assessment of key study variables.
- The data analytic approach appears appropriate and issues relating to power and attrition have been addressed.

Weaknesses

- Issues related to the blood draw that should be considered include the impact of diurnal variation in IL-6, resulting in a need to control time of day of sample collection.
- If examining markers of inflammation, factors that have an acute impact on circulating levels should be considered – e.g., acute illness, vaccination, antibiotics, anti-inflammatory medications. Group differences in other factors should be examined – e.g., inflammatory disease, medications that impact inflammatory mediators, adiposity.
- No methods are provided for the assessment of the proposed biomarkers. Evidence in support of reliability and validity of these methods is also needed.
- It is unclear whether the developed intervention, which appears to be adapted to meet the needs of elderly caregivers, is acceptably to younger caregivers.

5. Environment

Strengths

- Outstanding environment that will contribute to the success of the project.

Weaknesses

- None noted

Study Timeline

Strengths

- The study timeline has been carefully considered.

Weaknesses

- None noted

Protections for Human Subjects

Acceptable Risks and/or Adequate Protections

- No concerns noted.

Data and Safety Monitoring Plan (Applicable for Clinical Trials Only):

Acceptable

- The application has been modified to include a DSMB.

Inclusion Plans

- Sex/Gender: Distribution justified scientifically
- Race/Ethnicity: Distribution justified scientifically
- For NIH-Defined Phase III trials, Plans for valid design and analysis: Not applicable

- Inclusion/Exclusion Based on Age: Distribution justified scientifically
- No concerns noted.

Vertebrate Animals

Not Applicable (No Vertebrate Animals)

Biohazards

Not Applicable (No Biohazards)

Resubmission

- The application has been revised to address many of the issues that were raised. Revisions are satisfactory in regard to caregiving expertise of the team, the timeline, and concerns about the inclusion of both a pilot and a RCT trial. Preliminary data has been clarified and further evidence provided in support of the novelty of the approach and in regard to the analytic plan and sample size. As requested, the applicants have also added an independent DSMB.
- The resubmission includes a new conceptual framework. However, this model does not clearly address raised concerns about the role of biomarkers. A compelling rationale for examining HRV and blood markers as primary outcomes is lacking.
- It remains unclear how modifications from the ongoing pilot trial will inform the proposed trial.

Resource Sharing Plans

Acceptable

Budget and Period of Support

Budget Modifications Recommended (in amount/time)

Recommended budget modifications or possible overlap identified:

- The proposal seems top heavy, with many investigators and overlapping roles.

THE FOLLOWING SECTIONS WERE PREPARED BY THE SCIENTIFIC REVIEW OFFICER TO SUMMARIZE THE OUTCOME OF DISCUSSIONS OF THE REVIEW COMMITTEE, OR REVIEWERS' WRITTEN CRITIQUES, ON THE FOLLOWING ISSUES:

PROTECTION OF HUMAN SUBJECTS: ACCEPTABLE

INCLUSION OF WOMEN PLAN: ACCEPTABLE

INCLUSION OF MINORITIES PLAN: ACCEPTABLE

INCLUSION ACROSS THE LIFESPAN: ACCEPTABLE

COMMITTEE BUDGET RECOMMENDATIONS: Insufficient justification for the large number of investigators with overlapping roles

Footnotes for 1 R01 AG066081-01A1; PI Name: Mccrae, Christina S

Ad hoc or special section application percentiled against "Total CSR" base.

NIH has modified its policy regarding the receipt of resubmissions (amended applications).See Guide Notice NOT-OD-18-197 at https://grants.nih.gov/grants/guide/notice-files/NOT-OD-18-197.html. The impact/priority score is calculated after discussion of an application by averaging the overall scores (1-9) given by all voting reviewers on the committee and multiplying by 10. The criterion scores are submitted prior to the meeting by the individual reviewers assigned to an application, and are not discussed specifically at the review meeting or calculated into the overall impact score. Some applications also receive a percentile ranking. For details on the review process, see http://grants.nih.gov/grants/peer_review_process.htm#scoring.

MEETING ROSTER

The roster for this review meeting is displayed as an aggregated roster that includes reviewers from multiple CSR Special Emphasis Panels of the 202101 BBBP Aggregate for the 2021/01 council round.
This roster for CSR is available at:
http://public.era.nih.gov/pubroster/Reports?DOCTYPE=SEP&DESFORMAT=PDF&AGENDA_SEQ_NUM_P=407472
